# Combined primary hepatic neuroendocrine carcinoma and hepatocellular carcinoma: case report and literature review

**DOI:** 10.1186/s12957-021-02187-5

**Published:** 2021-03-16

**Authors:** Akira Nakano, Kenichi Hirabayashi, Hiroshi Yamamuro, Taro Mashiko, Yoshihito Masuoka, Seiichiro Yamamoto, Soji Ozawa, Toshio Nakagohri

**Affiliations:** 1 Department of Digestive Surgery, Tokai University Hospital, Shimokasuya 143, Isehara, Kanagawa 259-1143 Japan; 2 Department of Pathology, Tokai University Hospital, Shimokasuya 143, Isehara, Kanagawa 259-1143 Japan; 3 Department of Radiology, Tokai University Hospital, Shimokasuya 143, Isehara, Kanagawa 259-1143 Japan

**Keywords:** Neuroendocrine carcinoma, Hepatocellular carcinoma, Mixed neuroendocrine neoplasm

## Abstract

**Background:**

Hepatocellular carcinoma (HCC) can grow in a mosaic pattern, often combined with various non-hepatocellular cells. However, HCC combined with a neuroendocrine carcinoma (NEC) component is rarely reported, and its clinical features, origin, diagnosis, and behavior have not been established. In the literature, mixed HCC–NEC tumors are categorized as either collision type or combined type, depending on their microscopic features. Here, we report a patient with a combined-type HCC–NEC tumor.

**Case presentation:**

An asymptomatic 84-year-old woman was found to have a solid mass in the right lobe of the liver. Laboratory and radiologic examinations showed typical findings of HCC, including arterial-phase enhancement, and portal- and delay-phase washout. She was treated by partial laparoscopic hepatectomy of segment 5. Pathological examination showed that the tumor was predominantly HCC, partly admixed with an NEC component. A transitional zone between the HCC and NEC tissues was also observed. The tumor was finally diagnosed as a combined-type primary mixed NEC–HCC tumor. After the preoperative diagnosis, the patient underwent somatostatin receptor scintigraphy to detect the primary NEC lesion, but no accumulation was found in any other part of her body. She has been free of recurrence for 9 months since the surgery.

**Conclusion:**

Mixed HCC–NEC tumors are extremely rare, and correct diagnosis requires multidisciplinary collaboration. The accumulation of further cases is needed to help understand the exact pathology, diagnosis, and treatment of this disease.

## Background

Hepatocellular carcinoma (HCC) is the most common liver malignancy [1]. HCC often grows in a mosaic pattern, in which various cell types are arranged in different architectural patterns within a large tumor. HCC can occasionally combine with other non-hepatocellular cell types, of which the most common is cholangiocarcinoma [2]. In contrast, neuroendocrine carcinoma (NEC) in the liver is rare, and usually arises as a metastasis from other organs [3]. While primary hepatic neuroendocrine carcinoma (PHNEC) is particularly rare, combined primary HCC with PHNEC is even more rare. Combined PHNEC–HCC tumor histology is categorized into two types [4–6]: the collision-type tumor, in which two simultaneous but histologically distinct tumors are derived from the same organ with no histologic admixture, and the combined-type tumor, in which both components intermingle with each other and cannot be clearly separated in the transitional area within a single tumor nodule. Here, we present a patient with a combined PHNEC–HCC tumor with favorable prognosis and provide a literature review of reported cases.

## Case presentation

**Fig. 1:**
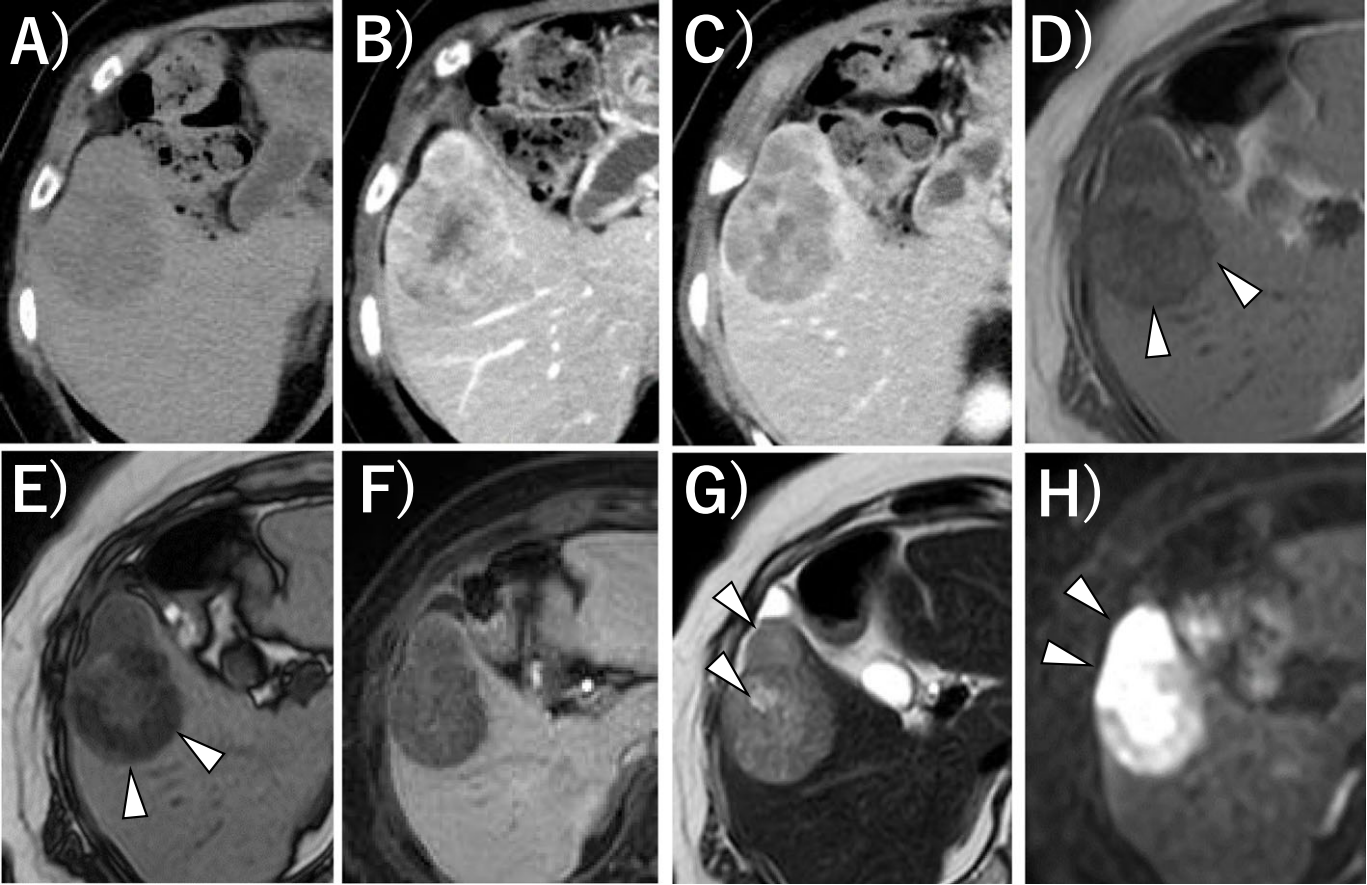
Preoperative CE-CT and EOB-MRI images. **a** Plain CT of the liver showed a 48×43 mm low-density lesion in the liver. **b**, **c** CE-CT showed contrast enhancement in the arterial phase (**b**) and hypoattenuation in the delayed phase (**c**). **d**–**h** EOB-MRI of the liver. The lower and peripheral parts of the lesion showed a lower signal in T1 weighted out-of-phase images (**e**) compared with in-phase images (**d**) (arrowhead). The lesion showed a low signal in the hepatobiliary phase (**f**). Fat-suppressed T2-weighted image (**g**) and diffusion-weighted image (**h**) showed a high signal in the upper and central parts of the lesion (arrowhead)

An 84-year-old Japanese woman with no history of hepatitis was referred to our institution because of a large solid mass in the right hepatic lobe that had been detected by computed tomography (CT) at another clinic. She had no specific symptoms, such as abdominal discomfort or jaundice. She was a non-smoker and a non-drinker, without obesity. The results of routine laboratory and liver function tests were within normal values. Her tests for serum hepatitis B surface antigen/antibody, hepatitis C antibody, and hepatitis C virus RNA were negative. Her serum alpha-fetoprotein level was high at 399 ng/mL (normal range, <20 ng/mL), and protein induced by vitamin K antagonist-II was slightly elevated at 43 mAU/mL (normal range, <40 mAU/mL). Other tumor markers, such as carcinoembryonic antigen and carbohydrate antigen19-9, were within their normal ranges. Plain CT revealed a single solid low-density mass at segment 5 in the liver. In contrast-enhanced CT, the lesion showed early hypervascularization and delayed hypoattenuation (Fig. 1a, b). Liver ethoxybenzyl diethylenetriamine pentaacetic acid-enhanced magnetic resonance imaging (EOB-MRI) showed some different features. The lower region and the periphery of the lesion showed low signals in T1-weighted out-of-phase images compared with in-phase images, which indicated that these parts of the lesion contained fatty tissue; this change was not observed in the left part of the lesion (upper and central parts on axial CT slice). Conversely, fat-suppressed T2-weighted and diffusion-weighted images showed high signals within the upper and central parts of the lesion. The patient was preoperatively diagnosed with HCC. Although the patient was old, her liver function was good (Child–Pugh class A), and there was only one lesion, and thus curative resection was considered possible. She underwent a laparoscopic partial hepatectomy of S5 and cholecystectomy without additional lymph node dissection. Because the tumor was located on the edge of the liver, the resected specimen was not large, measuring 80 mm.

**Fig. 2:**
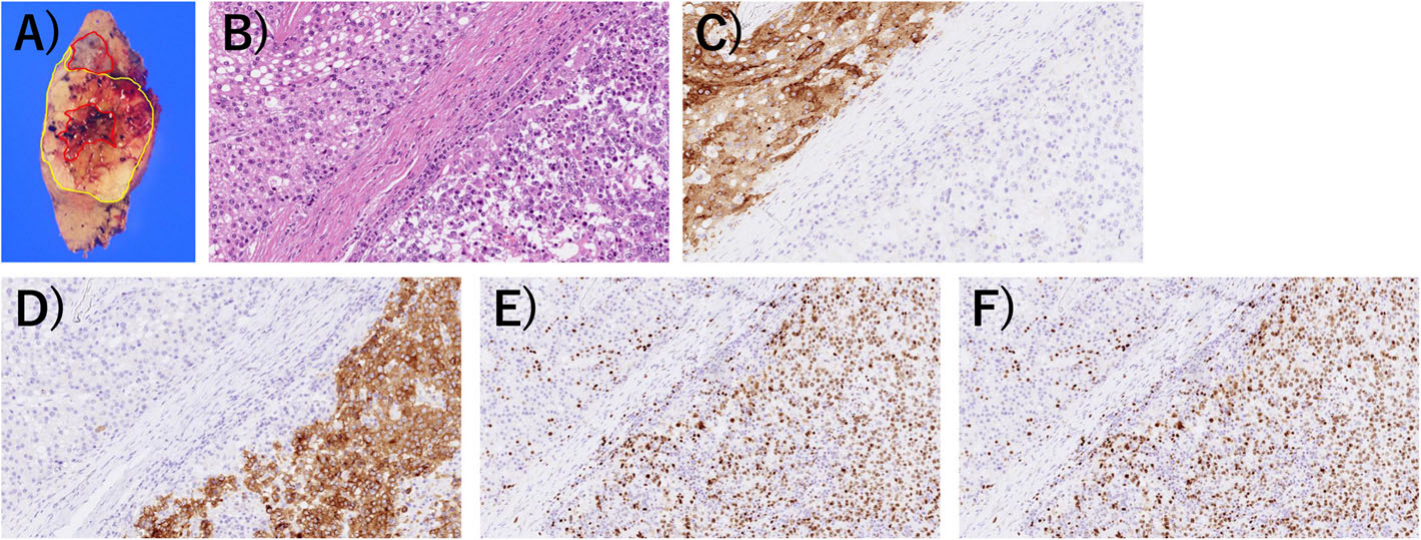
Macroscopic and microscopic findings of the tumor. **a** Tumor size was 55×45×40 mm, and the cut surface showed a predominantly HCC component (inside yellow line) admixed with NEC component (inside red line). **b** The HCC lesion (left upper side) and the NEC lesion (right lower side) (H&E stain). The HCC lesion was immunohistochemically positive for glypican-3 (**c**) but negative for synaptophysin (**d**) and chromogranin A (**e**). The NEC lesion was negative for glypican-3 (**c**) but negative for synaptophysin (**d**) and chromogranin A (**e**). The Ki-67 labeling index was 80% in the NEC lesion (**f**)

**Fig. 3:**
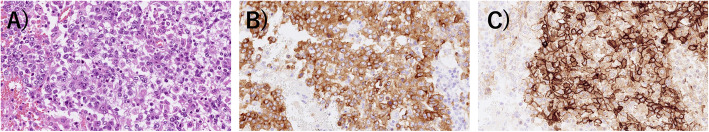
Microscopic findings of the transitional area between the HCC and NEC cells (**a**, H&E staining), which was positive for both synaptophysin (**b**) and glypican 3 (**c**)

Macroscopically, the tumor seemed solid, well-demarcated, and encapsulated, and was whitish-yellow in color with hemorrhagic and necrotic changes (Fig. 2a). Microscopically, the tumor showed both HCC and NEC components. The largest part of the tumor showed atypical cells with trabecularly arranged eosinophilic cytoplasm (Fig. 2b). Immunohistochemically, these atypical cells were diffusely positive for glypican-3 (Fig. 2c), but negative for chromogranin A and synaptophysin (Fig. 2d, e). The Ki-67 labeling index was approximately 10% (Fig. 2f). These histological and immunohistochemical features indicated moderately differentiated HCC. However, a grossly white-to-gray component was distributed from the center to the periphery of the tumor. This component was composed of a sheet-like arrangement of atypical cells with a high nucleus/cytoplasm ratio and many mitotic figures (Fig. 2b). The atypical cells in this area were immunohistochemically positive for synaptophysin and chromogranin A (Fig. 2d, e), but negative for glypican-3 (Fig. 2c). Their Ki-67 labeling index was approximately 80% (Fig. 2f). These histological and immunohistochemical features indicated NEC. The region of the tumor between the HCC and NEC components also showed atypical cells with enlarged nuclei and relatively broad cytoplasm (Fig. 3a), which were positive for both synaptophysin and glypican-3 (Fig. 3b, c), and seemed to be the transitional area between HCC and NEC. On the basis of these features and the absence of a primary NEC lesion outside of the liver, we diagnosed it as a combined PHNEC–HCC tumor.

The patient had a good postoperative course. One month after her surgery, she underwent somatostatin receptor scintigraphy to detect the primary NEC lesion. The examination showed no somatostatin accumulations in any other part of the body, which indicated that the NEC component was not metastatic. The patient was still alive 9 months after the surgery, with no sign of recurrence or metastasis.

## Discussion and conclusion

Primary hepatic NEC is a rare tumor, with an incidence of 0.3% among all neuroendocrine neoplasm (NEN) and 0.46% among primary hepatic malignancies [7]. As the liver is the most frequent site of NEN metastases from other organs, a systemic search for the primary lesion is necessary when hepatic NEN was suspected, considering the rarity of PHNEC. In contrast, HCC, the most common liver malignancy, often coexists with other malignancies. The most common of these combinations is HCC with intrahepatic cholangiocarcinoma, accounting for 2.0–3.6% of all primary hepatic malignancies [8].

Although the exact origin of PHNEC is unclear, two hypotheses have been proposed [9, 10]: (a) neuroendocrine cells in the intrahepatic bile duct epithelium undergo malignant conversion and become PHNEC, and (b) PHNEC originates from stem cells that have dedifferentiated from other malignant hepatic cells and convert into neuroendocrine cells. The latter concept can explain the lesions with different carcinomas at one site, whereas the former can only explain the pathogenesis of single PHNEC tumors.

In the literature, these composite liver tumors are classified as either collision type or combined type. In collision-type tumors, the HCC and NEC grow as distinctly separated compartments. In combined-type tumors, the HCC and NEC are closely intermingled and a transition zone can be found. These tumors used to be categorized as “mixed adeno-neuroendocrine carcinoma (MANEC)” when at least 30% of either component is identified [10]. However, with the revision of the World Health Organization (WHO) Classification in 2019, “collision type” tumors, in which two components appear to be independently derived and are simply adjoined with no observable mutual transition, were excluded from “mixed neuroendocrine neoplasm (MiNEN)” [11].

Concurrent occurrence of HCC and NEC is extremely rare. Including our patient, 21 English-language reports of 25 patients with HCC with NEC components have been published to date [4–7, 9, 12–26]. The clinicopathological profiles of these 25 patients are summarized in Table 1. These composite tumors were described as either collision type or combined type. In the present case, the two components were partly intermingled, and some portion of the HCC expressed neuroendocrine markers. Therefore, our case was classified as a combined-type MiNEN.

**Table 1:**
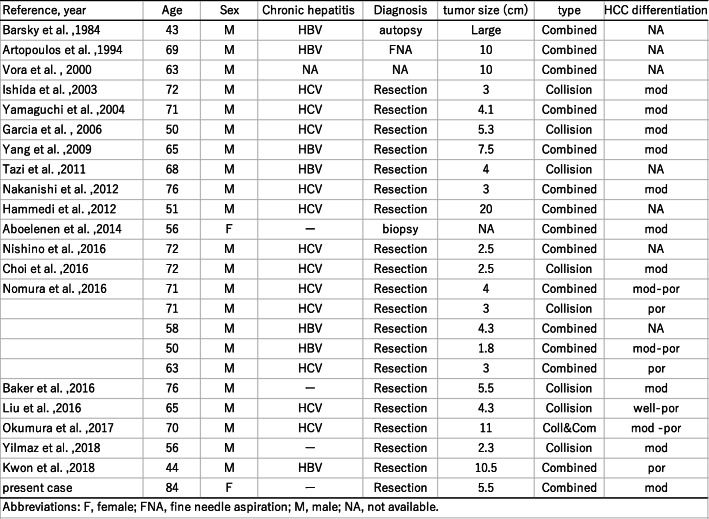
Clinicopathological profiles of 25 reported patients with HCC–NEC

Abbreviations: *F*, female; *FNA*, fine needle aspiration; *M*, male; *NA*, not available

In the literature, combined type (*n*=17) tumors are more common than collision type (*n*= 9; one patient had both collision- and combined-type tumors). Almost all the reported cases were preoperatively diagnosed as HCC and rediagnosed as combined or collision PHNEC–HCC tumors after resection, with only three cases diagnosed without surgery (by biopsy, fine needle aspiration, or autopsy); this demonstrates the difficulty of obtaining a correct preoperative diagnosis for these tumors.

In retrospect, the present case showed some interesting conformity between the preoperative images and the pathology. In the preoperative EOB-MRI, the lower and peripheral parts of the tumor showed a low signal in T1-weighted in- and out-of-phase images, indicating that this part contained fatty tissue and was thus highly suspicious of HCC. Furthermore, the upper and central parts showed extremely high signal intensity in the diffusion-weighted image (typical of NEC expression) just coinciding macroscopically with the distribution of HCC and NEC components in the tumor cut-surface, reflecting their mixture. Furthermore, the distributions of these components were morphologically presented as if the central part of the tumor had changed its character and had grown out to the ventral periphery. Previously, Yang et al. reported a combined-type tumor in which a poorly differentiated HCC focally expressed neuroendocrine marker [18], which resembled our case. In our case, these findings seem to support the supposition that a moderate or poorly differentiated HCC transdifferentiated into a neuroendocrine phenotype, resulting in a combined HCC–NEC tumor.

**Fig. 4:**
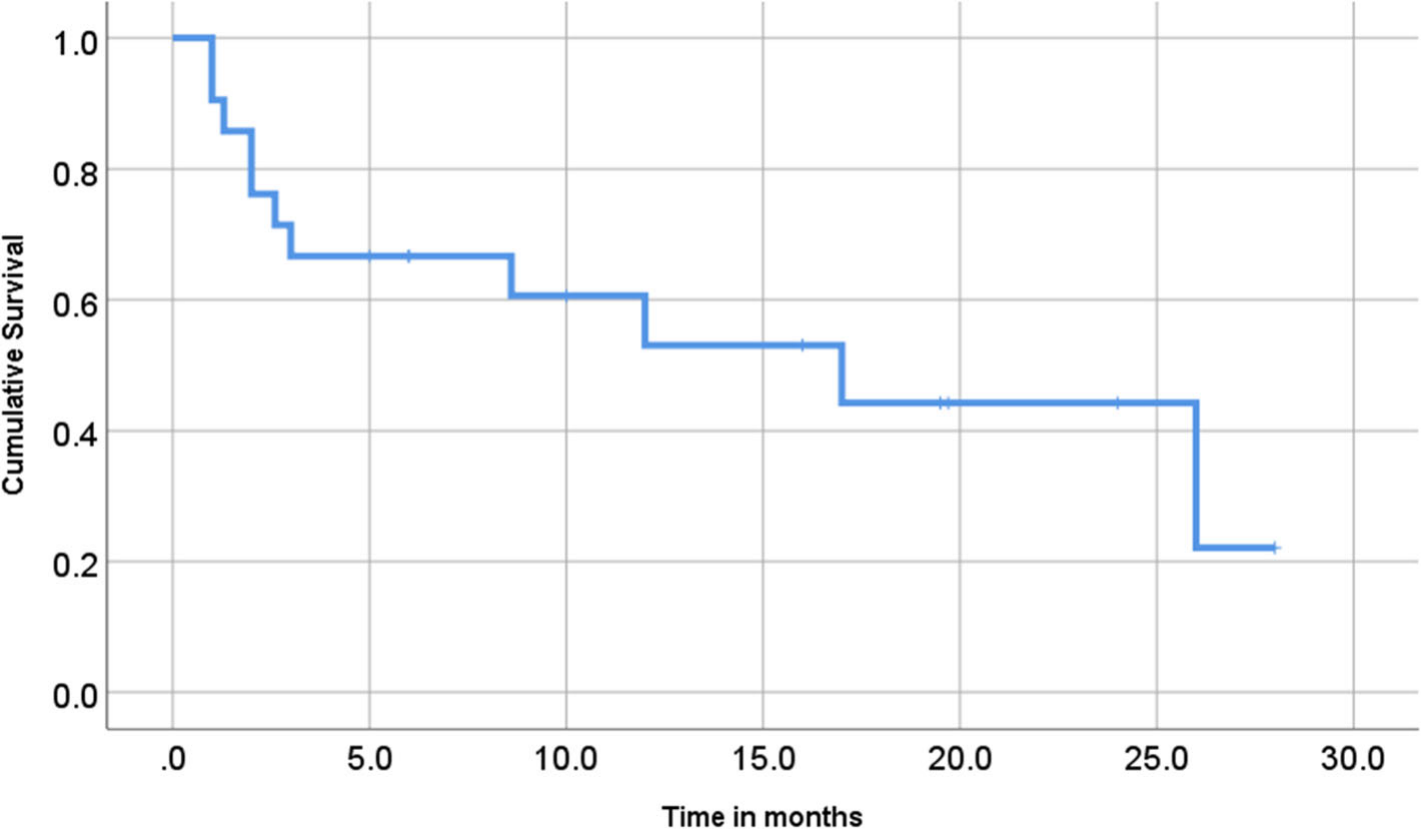
Cumulative survival of previously reported patients with HCC-PHNEC

The clinical significance of HCCs with NEC components is unclear. Several reports have shown that HCCs with NEC components are associated with aggressive behavior and dismal outcomes [4, 5]. Mixed PHNEC and HCC lesions tend to have a poor prognosis. Of the 25 cases summarized in this report, eight patients had recurrence, six patients died within a year after their surgeries, and only two patients were reportedly alive 2 years after surgery (Table 1). Although the number of reported cases is relatively small, the 1-year cumulative survival rate of the patients was 53% in our literature review (Fig. 4; prognosis not available for three patients). Among resected cases with recurrence or biopsy-confirmed metastasis, an NEC component was found in each case, which indicates that the NEC component behaved more aggressively than primary HCC, leading to a much poorer prognosis (Table 2). Therefore, identifying the neuroendocrine component is important for assuring that the patients can receive proper treatment. In our case, although patient was old, her liver was normal and without viral hepatitis or cirrhosis. We considered that surgery was a better treatment than tumor ablation or transcatheter arterial embolization (TAE) because the lesion was single, and curative resection can be easily achieved. As a result, it led to the histological diagnosis of this complex tumor.

**Table 2:**
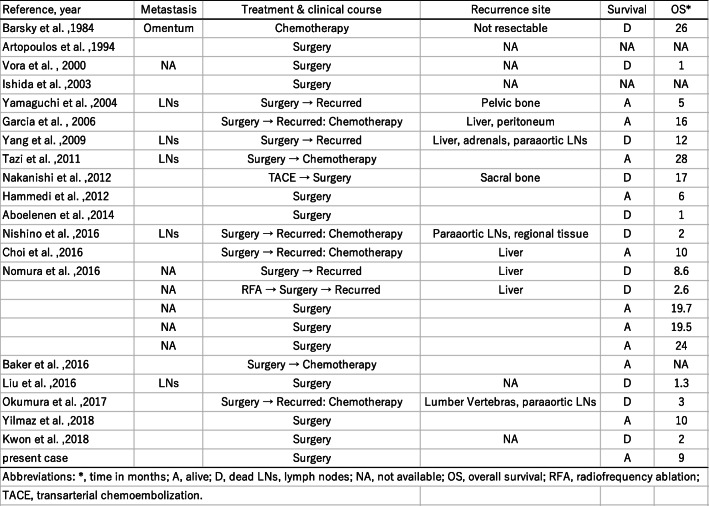
Treatment and prognosis of 25 reported patients with HCC–NEC

Abbreviations: *A*, alive; *D*, dead; *LNs*, lymph nodes; *NA*, not available; *OS*, overall survival; *RFA*, radiofrequency ablation; *TACE*, transarterial chemoembolization

*Time in months

In conclusion, mixed PHNEC and HCC tumors are extremely rare. Further accumulation of case reports is required to clarify the features, diagnostic details, and optimal therapy for combined PHNEC–HCC lesions.

## Data Availability

All data generated during this study are included in this published article.

## Abbreviations

CE-CT: Contrast-enhanced computed tomography
EOB-MRI: Ethoxybenzyl diethylenetriamine pentaacetic acid-enhanced magnetic resonance imaging
HCC: Hepatocellular carcinoma
MiNEN: Mixed neuroendocrine neoplasm
NEC: Neuroendocrine carcinoma
PHNEC: Primary hepatic neuroendocrine carcinoma
